# Equity implications of rice fortification: a modelling study from Nepal

**DOI:** 10.1017/S1368980020001020

**Published:** 2020-10

**Authors:** Naomi M Saville, Macharaja Maharjan, Dharma S Manandhar, Helen A Harris-Fry

**Affiliations:** 1 UN World Food Programme, Kathmandu, Nepal; 2 Institute for Global Health, University College London, London, WC1N 1EH, UK; 3 Mother and Infant Research Activities (MIRA), Kathmandu, Nepal; 4 London School of Hygiene and Tropical Medicine, WC1E 7HT, London, UK

**Keywords:** Rice fortification, Diets, Nutritional adequacy, Equity, Nepal

## Abstract

**Objective:**

To model the potential impact and equity impact of fortifying rice on nutritional adequacy of different subpopulations in Nepal.

**Design:**

Using 24-h dietary recall data and a household consumption survey, we estimated: rice intakes; probability of adequacy (PA) of eight micronutrients commonly fortified in rice (vitamin A, niacin (B_3_), pyridoxine (B_6_), cobalamin (B_12_), thiamin (B_1_), folate (B_9_), Fe and Zn) plus riboflavin (B_2_), vitamin C and Ca and mean probability of adequacy (MPA) of these micronutrients. We modelled: no fortification; fortification of purchased rice, averaged across all households and in rice-buying households only. We compared adequacy increases between population subgroups.

**Setting:**

(i) Dhanusha and Mahottari districts of Nepal (24-h recall) and (ii) all agro-ecological zones of Nepal (consumption data).

**Participants:**

(i) Pregnant women (*n* 128), mothers-in-law and male household heads; (ii) households (*n* 4360).

**Results:**

Unfortified diets were especially inadequate in vitamins B_12_, A, B_9,_ Zn and Fe. Fortification of purchased rice in rice-purchasing households increased PA > 0·9 for thiamin, niacin, B_6_, folate and Zn, but B_12_ and Fe remained inadequate even after fortification (PA range 0·3–0·9). Pregnant women’s increases exceeded men’s for thiamin, niacin, B_6_, folate and MPA; men had larger gains in vitamin A, B_12_ and Zn. Adequacy improved more in the hills (coefficient 0·08 (95 % CI 0·05, 0·10)) and mountains (coefficient 0·07 (95 % CI 0·01, 0·14)) but less in rural areas (coefficient −0·05 (95 % CI −0·09, −0·01)).

**Conclusions:**

Consumption of purchased fortified rice improves adequacy and gender equity of nutrient intake, especially in non-rice-growing areas.

Women’s diets are deficient in multiple micronutrients across many low-income settings^(1)^, and these deficiencies have negative effects throughout the life course. Before and during pregnancy, inadequate micronutrient intake adversely affects the risk of maternal mortality, offspring’s fetal growth and birth weight^(2,3)^. Despite political commitments to address malnutrition, at least two billion people suffer from micronutrient deficiencies globally^(4)^.

Nepal Demographic Health Surveys estimate that anaemia has risen to 41 % in women of reproductive age in recent years. This is particularly concerning because anaemia is a major cause of maternal mortality^(5)^. Beyond anaemia, deficiencies of multiple micronutrients are prevalent in Nepal and exist concurrently. Amongst pregnant women in the *Terai*, Jiang *et al.* ^(6)^ found micronutrient deficiencies to be common, including vitamins A (7 %), E (25 %), D (14 %), riboflavin (33 %), vitamin B_6_ (40 %), B_12_ (28 %), folate (12 %), Zn (61 %) and Fe (40 %). The 2016 Nepal National Micronutrient Status Survey reported low ferritin in 27·6 % of children aged 6–59 months, 18·7 % of non-pregnant – and 14·2 % of pregnant women^(7)^. Meanwhile, Zn and vitamin A deficiencies were detected in 20·7 and 4·2 % of children aged 6–59 months and 24·3 and 3 % of non-pregnant women, respectively^(7)^.

Limited dietary data from Nepal indicate that diets fail to provide adequate nutrients^(8,9)^. In rural mountainous areas, 34 % of people were both food poor (unable to access sufficient energy) and nutrient poor (unable to access sufficient micronutrients), while 24 % were nutrient poor only^(10)^. Across Nepal, rice is consumed daily, in large quantities^(8,11)^, and forms the major source of micronutrients^(9,11)^. Realistic dietary recommendations based on locally available foods may be insufficient to meet nutritional gaps^(12)^, and food-insecure households are unlikely to achieve nutritional adequacy by this means in the short term^(13,14)^.

Fortification of rice in Asia has emerged as a means to improve nutritional adequacy^(15–20)^. In Bangladesh, fortification of rice through social safety nets is having very positive results^(21)^. In Cambodia, fortified rice school meals had positive effects on cognition^(22)^ and micronutrient status^(23)^, although effects were limited by inflammation^(24)^. Fe fortification of rice decreased anaemia significantly amongst school children in Bangalore^(25)^, Andhra Pradesh^(26)^, Odisha^(27)^, Brazil^(28,29)^ and the Philippines^(30)^ and factory workers in Mexico^(31)^. In Costa Rica, mandatory rice fortification, together with other fortification efforts, was associated with decreased anaemia, B_9_ (folate) deficiency and neural tube defects^(31,32)^.

With this in mind, rice fortification in Nepal has been identified as a potential strategy to reduce micronutrient deficiencies. It can deliver micronutrients safely and cheaply, builds on existing infrastructure and requires minimal behavioural change^(18,33)^. The Government of Nepal, in partnership with the UN World Food Programme (WFP), intends to introduce fortified rice via Nepal Food Corporation (NFC) social safety nets in remote and food-insecure districts, by establishing blending units in NFC rice mills. Later, introduction of blending technology to private sector mills will enable wider distribution of fortified rice. However, Nepalese rice intakes, dietary nutrient deficiencies and the potential for rice fortification to address them have not been explored. Whilst increased adequacy of fortified nutrients is expected with fortification, measurement of potential differential impacts in population subgroups is needed to examine who will benefit most from rice fortification in Nepal. Moreover, whilst some studies have modelled the micronutrient contribution of fortified foods, they have failed to account for total dietary intake^(34)^. Thus, in the current study, we model the potential impacts of rice fortification on nutritional adequacy of the entire diet of different population subgroups to investigate the equity impact and inform rice fortification in Nepal.

## Methods

Between June and September 2015, we collected individual 24-h dietary recalls of pregnant women, their mothers-in-law and male household heads, in Dhanusha and Mahottari districts in province 2^(35–39)^, as part of the Low Birth Weight South Asia Trial (LBWSAT)^(40,41)^. Between 19 September 2014 and 16 July 2015, the third Nepal Annual Household Survey (AHS III) estimated 7-d household food consumption for a nationally representative sample of households^(42)^. These two studies provide rich data to estimate the potentially differential benefits of rice fortification in different population subgroups. Using LBWSAT individual-level dietary data, we can precisely model the potential impacts of rice fortification and estimate the intra-household equity implications of rice fortification in province 2, where underweight and anaemia are highest^(43)^. Using AHS III data provides a national picture of consumption and enables us to model potential rice fortification impacts on adults and children, and in different geographies.

### Sampling and field procedures

The LBWSAT protocol^(40,44)^ and dietary measures^(35,36)^ have been described elsewhere. Whilst 24-h dietary recalls were collected in 805 households in eighty Village Development Committee clusters overall, we use data from 128 households sampled in the twenty control clusters. Within each traditional joint, male-headed household sampled, dietary recalls were taken with the pregnant woman, her mother-in-law and male household head. Recalls were repeated up to three times per person on non-consecutive days within a period of 2 weeks^(35,36)^. Portion sizes were estimated using a pictorial food atlas of portion sizes that was developed and validated locally^(36)^.

The AHS III sampled fifteen households in each of 288 primary sampling units that were selected using population proportional to size from 4861 urban and 36 191 rural enumeration areas. Selected primary sampling units covered sixty-five of the seventy-five districts in Nepal and thus may be considered to be nationally representative, covering all agro-ecological zones and wealth groups. Using 2011 household census lists, 4360 households were randomly selected for interview. After describing the household composition using a roster, respondents were interviewed about their consumption of a list of foods and drinks, yielding a dataset with the details of consumption of sixty foods. If an item was consumed in the preceding week, enumerators recorded the frequency (d/week) and quantity (kg/week from home production, purchase or receipt in-kind) that the item was consumed^(42)^.

### Analysis of dietary data

In both datasets, we calculated the proportion of households consuming rice and purchased rice, mean rice consumption (g/d) for each household member. With the AHS III, we calculated mean intake of purchased rice and used adult male equivalents (AME)^(45)^ to allocate household-level intakes to individuals. We calculated average daily nutrient intakes using nutrient composition values from a Nepal-specific food composition table (FCT) (available on request)^(36)^.

We estimated nutritional adequacy of eight micronutrients normally recommended for rice fortification^(46)^: vitamins A, B_3_ (niacin), B_6_ (pyridoxine), B_12_ (cobalamin), B_1_ (thiamin), B_9_ (folate), Fe and Zn, plus three other micronutrients: Ca, B_2_ (riboflavin) and vitamin C. Then, by substituting micronutrient values of unfortified rice with the WFP 2016 fortified rice specification^(47)^, we quantified the potential increase from rice fortification in probability of adequacy (PA) for each nutrient, mean probability of adequacy (MPA) of eleven nutrients and equity of nutrient adequacy.

Table 1 provides the nutrient levels in unfortified uncooked rice, the estimated levels of water-soluble B vitamins in unfortified cooked rice after applying a conversion factor of 1/0·36 = 2·78 for deriving dry weight, the WFP^(47)^ and Bangladesh^(48)^ fortified rice specifications and those recommended by DePee *et al.* ^(49)^. Despite evidence of nutrient losses during washing and cooking of milled rice^(50)^, we were not able to access estimates of losses in the Nepal context, where cooking method varies by region. Hence, we used uncooked rice nutrient estimates, since the Nepal^(51)^ and India^(52)^ FCT provide only uncooked rice, and FCT vary widely in their rice estimates. Also, AHS III estimated dry uncooked rice consumption only, so introducing conversions to cooked rice would have introduced further error. Table 1 shows the figures used for uncooked rice and two alternative estimates of water-soluble B vitamins in cooked rice (converted to the dry weight) from figures in the Bangladesh^(53)^ and United States Department Agriculture^(54)^ FCT. For B_6_ and B_3_, our raw rice estimates lie in the estimates of cooked rice from United States Department Agriculture and Bangladesh FCT, whereas for B_1_, B_2_ and B_9_, the cooked values are somewhat lower than those for raw rice.

**Table 1:** Micronutrient levels in unfortified uncooked rice and in dry weight of cooked rice from various sources

Vitamin	Parameters	Unit	Per 100 g uncooked rice	FCT	Bangladesh estimates: water-soluble B vitamins/100 g dry weight*		USDA estimates: water-soluble B vitamins/100 g dry weight*		WFP standard: fortified uncooked rice†	Bangladesh* standard: fortified uncooked rice‡	de Pee *et al.* ^(49)^ recommendation: fortified uncooked rice§
	Energy	kJ	1443	Nepal							
	Protein	g	6·8	Nepal							
	Ca	mg	10	Nepal							
	Se	mcg	1·01	India							
	Zn	mg	1·21	India					6·5	4·0	6·0
	Fe	mg	0·7	Nepal					4·5	6·0	4·0
A	Vitamin A	RE (mcg)	0	India					175	183	150
B_1_	Thiamin	mg	0·21	Nepal	0·03 × 2·78 =	0·08	0·02 × 2·78 =	0·06	0·75	0·5	0·5
B_2_	Riboflavin	mg	0·06	Nepal	0·01 × 2·78 =	0·03	0·01 × 2·78 =	0·03			
B_3_	Niacin	mg	1·9	Nepal	1·1 × 2·78 =	3·06	0·04 × 2·78 =	0·11	8·5	0	7·0
B_6_	Pyridoxine	mg	0·12	India	0·03 × 2·78 =	0·08	0·09 × 2·78 =	0·25	0·9	0	0·6
B_9_	Folate	mcg	9·32	India	3 × 2·78 =	8·34	3 × 2·78 =	8·34	317	267	
	Folic acid	mcg							190	160	130
B_12_	Cobalamin	mcg	0	India	No info	No info	0	0	1·5	1·2	1·0
C	Ascorbic acid	mg	0	India							
E	α-Tocopherol	mg	0·06	India							

FCT, Food Composition Table; WFP, World Food Programme; USDA, United States Department Agriculture; RE, retinol equivalents.

* The conversion factor for weight of uncooked to cooked rice is 0·36 making the conversion from cooked rice to dry rice 1/36 = 0·278.

† WFP 2016 specification based on mid-point between minimum and maximum levels leaving factory.

‡ Bangladesh estimates are for the household level after losses from storage.

§ For population consuming 150–300 g/d^(49)^.

Since only purchased rice is going to be fortifiable in the foreseeable future, we modelled fortification of purchased rice only, not home-grown rice. For LBWSAT, we estimated the purchased share of rice based on ranking (1–5) of the importance of purchasing as their main rice source, assuming that the ranking was proportional (1 = 100 % of rice bought; 2 = 80 % bought, and so on).

PA was calculated using the ‘probability approach’^(55)^. For LBWSAT, we estimated daily ‘usual’ intakes by transforming nutrient intakes to normal distributions using Box–Cox transformations^(56)^ and calculating the best linear unbiased predictors to account for within- and between-person variance. For AHS III, daily usual intakes were simply 1/7 of weekly intakes. For both datasets, we calculated PA by comparing intakes (apart from Fe for non-pregnant women) with known nutrient requirement means (estimated average requirements (EAR)) and sd ^(57–60)^. We assumed low bioavailability of Fe (at 5 %, apart from pregnant women at 23 %)^(57)^ and Zn (18 % men; 25 % women; 23 % children)^(61)^. When unavailable, we calculated EAR for all age groups and pregnancy status using reference nutrient intakes for each nutrient as follows: EAR = reference nutrient intake ×((2 × CV/100) + 1), and sd as EAR × CV. We used US Institute of Medicine values for Ca (EAR and sd)^(58)^ and Fe (PA)^(59)^. For Zn, we used EAR from the IZiNCG study^(61)^, and for all other nutrients, we used FAO/WHO 2004 values^(57)^ based on estimates of CV from WHO 2006^(62)^.

### Sensitivity analyses

As a sensitivity analysis of different fortificant blends, we repeated these analyses using the Bangladesh fortified rice standard. We did not model the Indian standard as this is unlikely to be used in Nepal due to its riboflavin content, which affects colour, flavour and acceptability^(63)^. As LBWSAT data were collected during mango season, we report vitamin A intakes with and without mangos. AHS III data were missing green leafy vegetables and eggs, so we conducted a sensitivity analysis with a daily 50 g portion of green leafy vegetables, and one egg added to the diet.

Since the AHS III analyses rely on AME to estimate individual intakes, we used LBWSAT data to measure the agreement between observed and predicted intakes for pregnant women, their mothers-in-laws and male household heads with and without adjustments for pregnancy, physical activity levels and body weight. The Bland–Altman limits of agreement were calculated as the mean kJ difference between observed and predicted intakes (sd 1·96)^(64)^.

We also calculated the percentage whose micronutrient intake exceeded upper tolerable limits of the fortifiable nutrients^(57)^.

### Equity analyses

To estimate the potential gender equity impact of fortification in LBWSAT, we calculated the increase in PA from fortification for pregnant women and men and differences between the two (difference in difference). Then, we tested whether the intercept of this difference in difference was significantly different from zero using linear regression models. To estimate the differential impact of fortification by regions in AHS III, we applied double-hurdle regressions (Cragg’s models) which account for the large number of zeros for people who have no fortification because they are home producers. The models assess the probability of benefiting from fortification in two equations (i) whether or not households have any improvements in MPA (determined by whether they purchase any rice) and (ii) how much the MPA improved (determined by how much purchased rice is consumed). We report the conditional mean estimates of the mean difference in PA comparing all other provinces with province 3 (where Kathmandu is based), hills and mountains with *Terai* and rural with urban areas. All analyses and reported se account for survey design and were conducted in Stata SE 14 (StataCorp LP).

## Results

### Response rate and respondent characteristics

The LBWSAT sample included 150 households out of 199 eligible households visited, and we modelled potential effects of rice fortification on 128 households (1230 dietary recalls from 384 individuals) because twenty-two households had missing information on rice purchase. Prior analyses show characteristics of respondents and non-respondents were similar^(38)^. AHS III data contain 4360 households with 5443 women aged 15–49 years and 3346 children aged 5–12 years.

Characteristics of the households are given in Table 2.

**Table 2:** Socio-demographic and health characteristics of the samples

LBWSAT				National AHS III*			
	*n*	Median†, mean‡ or %	25th, 75th centile		*n*	Median†, mean‡ or %	25th, 75th percentiles or (se)
Age				Age			
Pregnant women†	150	21	19, 24	Women 15–49 years	5443	28	21, 36
Mothers-in-law†	150	50	44, 56	Children 5–12 years	3346	9	7, 11
Male HH heads†	150	39	25, 56				
Caste group	150						
Disadvantaged§		35·3 %					
Middle‖		42·7 %					
Least disadvantaged¶		22·0 %					
Maternal education	150			Maternal education			
No schooling		56·1 %		Ability to read (%)	4360	49·6 %	
Primary class 1–8		27·0 %		Education grade	4360	3·4	(0·2)
Secondary class 9 and above		16·9 %					
Male education	147			Male education			
No schooling		42·2 %		Ability to read (%)	4360	68·6 %	
Primary class 1 to 8		29·9 %		Education grade	4360	5	(0·1)
Secondary class 9 and above		27·9 %					
Overseas migration	128						
Any household member		46·1 %					
Household Food Insecurity Access Scale	134						
Any food insecurity**		30·6 %					
Minimum dietary diversity††	150						
Pregnant women		58·0 %					
Mothers-in-law		59·3 %					
Male HH heads		62·0 %					
Low mid-upper arm circumference (<23 cm)	150						
Pregnant women		40·0 %					
Mothers-in-law		35·3 %					
Male HH heads		14·0 %					

AHS III, the third Nepal Annual Household Survey; LBWSAT, Low Birth Weight South Asia Trial; Male HH heads, male household heads.

* Estimates after applying sampling weights.

† Median 25th and 75th percentiles.

‡ Mean and se.

§ Disadvantaged: Dalit and Muslim.

‖ Middle: *Janjati*/other *Terai* castes.

¶ Least disadvantaged: *Yadav*, *Brahmin*.

** Food security recall in the past 4 weeks.

†† Adequate dietary diversity ≥5 out of ten food groups.

In both LBWSAT and AHS III samples, women and men are poorly educated. Pregnant women from LBWSAT are younger (median age 21 years), and mothers-in-law are older (50 years) than the women of reproductive age in the AHS III sample (28 years). Nearly half of LBWSAT households have members working overseas, and perceived food insecurity and thinness (mid-upper arm circumference <23 cm)^(65,66)^ are prevalent, the latter particularly amongst pregnant women. Diets lack diversity with 38–42 % consuming fewer than the recommended five out of ten food groups on the 1st day of recall^(67)^.

### Rice intakes

**Fig. 1:**
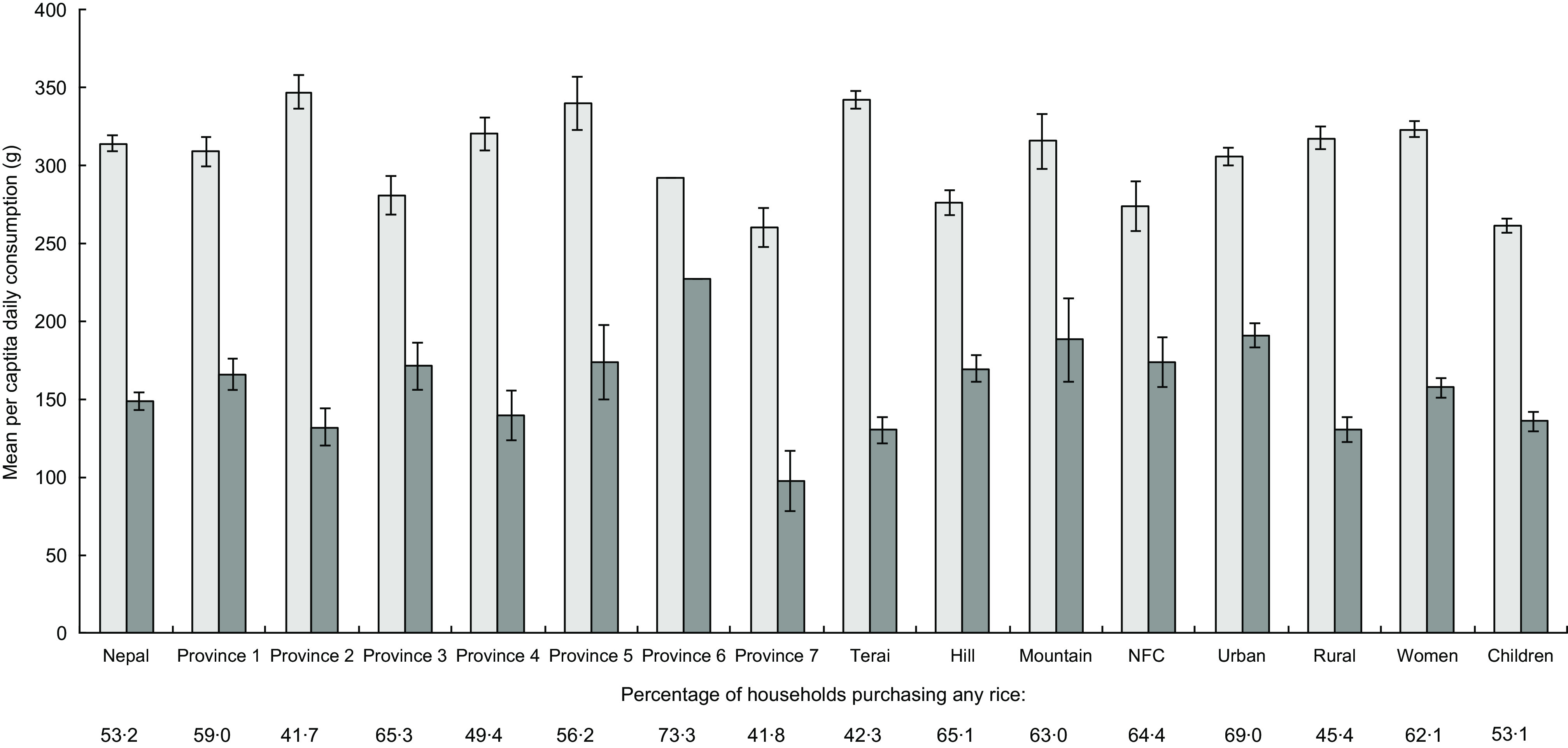
Mean intakes of all rice and purchased rice by province, ecological zone, rural/urban, women and children. Using Annual Household Survey 2014–15 data. Percentage of households purchasing rice is provided below each category. 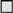, rice from all sources (purchased, grown, received); 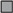, purchased rice only (potential for fortification). NFC, Nepal Food Corporation

The LBWSAT plains population (see online supplementary material, Supplemental Table 1) and AHS III Nepal-wide (Fig. 1) intake data show that rice is consumed regularly, by most people, and in high quantities, making it a promising candidate for fortification.

In LBWSAT, boiled white rice made up the majority of the diet. Almost all (98 %) households ate rice over the 3 d of dietary recall, and 76 % purchased it. Respondents consumed rice 1·8 times/d amounting to a median cooked weight of 667 g/d. Fried rice, rice pudding, porridge (‘khichadi’ or ‘jaulo’), beaten rice, puffed rice or as an ingredient in deep-fried snacks were occasionally consumed, but the mean frequencies of consumption (range 0·01–0·10 times/d) were much lower than boiled rice. Wheat, consumed as an unleavened bread called ‘roti’, and ‘dal’ (lentil soup) were both consumed on average once per day.

Consistent with LBWSAT results, 99 % of AHS III households consumed rice in the preceding week (Fig. 1).

Per capita daily uncooked rice consumption was lower than LBWSAT figures, at mean 314(sd 170) g/d and 149 (sd 186) g/d of purchased rice. Whilst overall rice consumption was highest in the *Terai* province 2 (where LBWSAT was located), the results indicate that the mountains and urban areas would benefit most from fortification, since the consumption of purchased rice was highest in these provinces (provinces 6, 5, 3 and 1).

### Current micronutrient intakes and adequacy

In both samples, micronutrient intakes before fortification (‘current’ rows in Table 3) show diets are low in key micronutrients. Despite the different methods of estimation, micronutrient intakes were reasonably similar between LBWSAT and AHS III, although intakes were lower in AHS (see Table 3 ‘current’). In LBWSAT, nutrient intakes were highest amongst men, followed by pregnant women, and mothers-in-law ate least.

**Table 3:** Mean dietary nutrient intakes, with and without fortification of purchased rice*

	National AHS III				LBWSAT					
	Women 15–49 years		Children 5–12 years		Pregnant women		Mothers-in-law		Male HH heads	
*n*										
Current	5443		3346		128		128		128	
Fortified (all)	5443		3346		128		128		128	
Fortified (buyers)	3007		1882		97		97		97	
Nutrients not in rice premix	Mean	sd	Mean	sd	Mean	sd	Mean	sd	Mean	sd
Energy (kJ/d)										
Current	8619	3130	6883	2644	8891	3050	8824	3268	11 263	3201
Protein (g/d)										
Current	60·6	24·9	47·2	20	63·1	24	62·8	27·4	80	25·5
Vitamin C (mg/d)										
Current	57·6	49·7	41·3	36·6	124·6	152·6	130·8	131·5	124·4	100·6
Ca (mg/d)										
Current	303	234	236	184	548	422	448	281	594	417
Vitamin B_2_ (mg/d)										
Current	0·7	0·4	0·6	0·3	1·1	0·6	1	0·6	1·2	0·6
Nutrients in rice premix	Mean	sd	Mean	sd	Mean	sd	Mean	sd	Mean	sd
Vitamin B_3_ (mg/d)										
Current	16·7	7·2	13·1	6	17·4	6·3	17·8	8	22·9	7·7
Fortified (all)	30·1	18·2	24·6	15·8	31·4	14·3	31·4	15	39·5	16
Fortified (buyers)	42·7	16·5	34·9	14·9	35·6	13·8	35·5	14·5	44·4	14·6
Vitamin B_6_ (mg/d)										
Current	2·2	0·9	1·7	0·7	2·2	0·9	2·2	0·9	2·8	0·9
Fortified (all)	3·6	2	2·9	1·7	3·7	1·7	3·7	1·6	4·5	1·8
Fortified (buyers)	4·9	1·9	4	1·7	4·2	1·7	4·1	1·6	5·1	1·7
Vitamin A (RE/d)† ‡										
Current	328	487	242	359	477	444	531	687	504	411
Fortified (all)	603	568	480	437	766	520	812	766	845	483
Fortified (buyers)	836	512	658	407	849	545	887	830	917	480
Vitamin B_1_ (mg/d)†										
Current	1·7	0·7	1·3	0·6	1·8	0·7	1·8	0·8	2·3	0·9
Fortified (all)	2·9	1·6	2·3	1·4	3·1	1·3	3	1·4	3·8	1·5
Fortified (buyers)	3·9	1·5	3·2	1·4	3·4	1·3	3·4	1·3	4·2	1·4
Vitamin B_9_ (μg/d)†										
Current	265	167	200	121	326	163	341	170	390	153
Fortified (all)	763	616	630	527	851	471	850	459	1010	498
Fortified (buyers)	1240	522	1013	459	1013	422	1000	415	1183	432
Vitamin B_12_ (μg/d)†										
Current	0·5	0·6	0·4	0·4	0·7	0·8	0·6	2·3	0·7	1·1
Fortified (all)	2·8	2·8	2·4	2·4	3·2	2·2	3	3·3	3·7	2·5
Fortified (buyers)	5·1	2·3	4·2	2	3·9	2	3·9	3·4	4·6	2·1
Fe (mg/d)†										
Current	11·7	5·4	9·2	4·4	15	5·6	15·3	7·3	19·6	6·5
Fortified (all)	18·8	10·2	15·3	8·7	22·4	8·8	22·5	10·2	28·4	9·6
Fortified (buyers)	25·3	9·5	20·7	8·3	24·5	8·7	24·7	10·1	30·9	9·2
Zn (mg/d)†										
Current	9·5	3·9	7·5	3·2	10	3·6	10	4·5	12·8	4·3
Fortified (all)	19·7	13·1	16·3	11·4	20·8	10·2	20·5	10·5	25·5	11·4
Fortified (buyers)	29·4	11·6	24·1	10·5	24	9·6	23·7	9·8	29·4	10·1

AHS III, the third Nepal Annual Household Survey; LBWSAT, Low Birth Weight South Asia Trial; Male HH heads, male household heads; Current, current intakes of total population with no fortification; Fortified (all), intakes of total population when bought rice is fortified with World Food Programme median values; Fortified (buyers), intakes of rice-buying households only, when bought rice is fortified; WFP, World Food Programme.

* Fortification using WFP specifications.

† Nutrient is also fortified in Bangladeshi rice premix, as reported in online Supplemental Table 2.

‡ Vitamin A intakes excluding mango are: pregnant women 334 ± 312 RE/d; mothers-in-law 360 ± 644 RE/d; male household heads 353 ± 336 RE/d.

The micronutrients that are consumed in particularly low levels are those that are not found in unfortified rice. Dehulled, non-parboiled, non-fortified rice provides no vitamins A or B_12_, very little of vitamins B_1_ (thiamin), B_2_ (riboflavin), Fe, Zn, vitamin B_6_ (pyridoxine) and somewhat higher levels of B_9_ (folate) and B_3_ (niacin) (see Table 1 and de Pee^(46)^). Our sensitivity analysis of adding a daily portion of 50 g green leafy vegetables and one egg to AHS estimates generated intakes more similar to LBWSAT. Vitamin B_12_ intakes were close to zero for all subgroups.

**Fig. 2:**
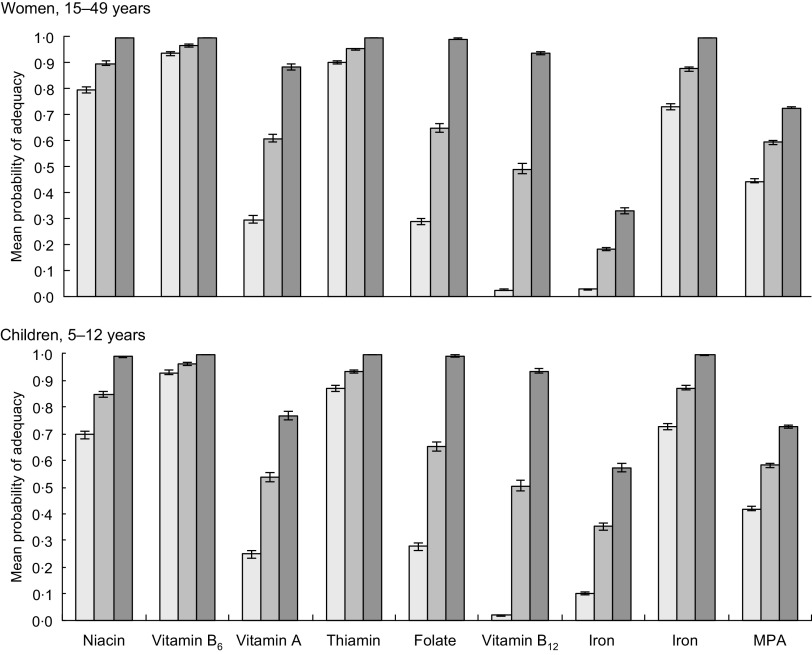
Probability of micronutrient adequacy of women and children in Nepal, with and without rice fortification. Using Annual Household Survey 2014–15 data. Current: based on unfortified diets of total population (women *n* 5443; children *n* 3346). Fortified (all): based on total population when bought rice is fortified with World Food Programme (WFP) mid-point values (women *n* 5443; children *n* 3346). Fortified (buyers): based on intakes of rice-buying households only, when bought rice is fortified with WFP mid-point values (women *n* 3007; children *n* 1882). 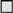, current; 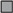, full sample; 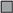, buyers only. MPA, mean probability of adequacy

**Fig. 3:**
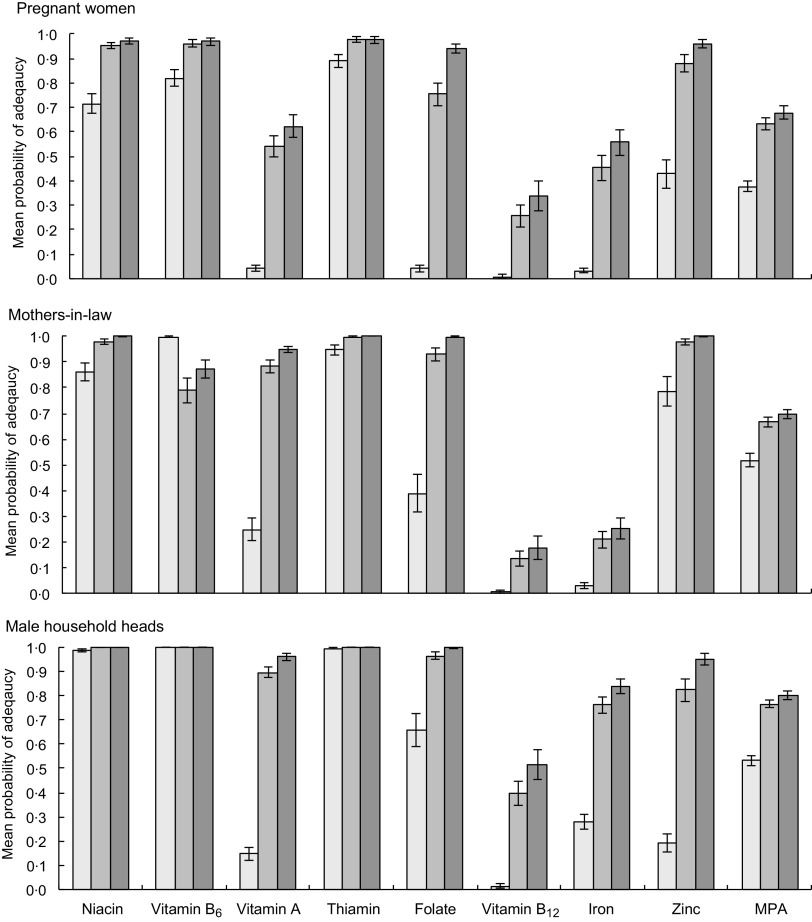
Probability of micronutrient adequacy of pregnant women, their mothers-in-law and male household head in Nepal province 2, with and without rice fortification. From Low Birth Weight South Asia Trial data. Current: based on unfortified diets of all respondents (*n* 128); Fortified (all): based on intakes of all respondents when bought rice is fortified with World Food Programme (WFP) mid-point values (*n* 128); Fortified (buyers): based on intakes of rice-buying households only, when bought rice is fortified with WFP mid-point values (*n* 97). 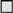, current; 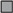, full sample; 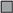, buyers only. MPA, mean probability of adequacy

The PA in the unfortified diets is shown in the ‘current’ bars in Fig. 2 (AHS III) and Fig. 3 (LBWSAT).

Across both samples, diets with unfortified rice are highly deficient in multiple micronutrients, notably vitamins B_12,_ B_9_ (folate) and A, Fe and Zn for all household members studied. In the unfortified diets of pregnant women, only vitamins B_3_ (niacin), B_6_ (pyridoxine) and B_1_ (thiamin) exceeded 50 % PA, while vitamins A, B_9_ (folate), Fe and Zn all lie below 10 % PA and vitamin B_12_ has a PA approaching zero (Fig. 3). Whilst deficiencies in the diet are less extreme for male household heads and mothers-in-law, PA below 30 % are found for vitamin A, Fe, Zn, and PA for vitamin B_12_ is, again, close to zero. Similarly, unfortified AHS diets are highly deficient in vitamins B_12_, A, B_9_ (folate) and Fe.

### Potential micronutrient intakes and adequacy after rice fortification

All population subgroups analysed show large increases in micronutrient intakes after fortification, with much higher increases when only rice-purchasing households are analysed. Table 3 provides daily nutrient intakes when all purchased rice in the diets is fortified, averaged across all households (labelled ‘Fortified (all)’) and in rice-purchasing households (labelled ‘Fortified (buyers)’). This shows how potential effects will be constrained by households who do not purchase rice and therefore do not benefit from fortification. Fortification with the Bangladesh standard premix (see online supplementary material, Supplemental Table 2) leads to broadly similar, although slightly lower, intakes than consumption of the WFP standard except for B_3_ (niacin) and B_6_ (pyridoxine), which are not in the Bangladesh standard.

The PA in the ‘current’, ‘Fortified (all)’ and ‘Fortified (buyers)’ diets is given for AHS women and children (Fig. 2) and LBWSAT household members (Fig. 3). After substituting normal rice with fortified rice using fortificant levels in the 2016 WFP specifications, dietary deficiencies of key nutrients are largely resolved in rice-purchasing households. Smaller but nevertheless substantial improvements in mean adequacy levels are found when non-rice purchasing households (who would not access fortified rice) are included in the population average. Amongst rice purchasers in all population subgroups, PA exceeded 90 % for B_3_ (niacin), B_6_ (pyridoxine), B_1_ (thiamin), B_9_ (folate) and Zn after fortification. Vitamin A increased to >85 % PA for AHS women, male household heads and mothers-in-laws but was lower for pregnant women and children. After fortification of rice, the mean PA of Fe did not reach 100 % for any household member category. The impact of fortified rice on Fe adequacy was lowest for mothers-in-law, reflecting their lower rice consumption than other family members. However, if we assume that Fe bioavailability is higher due to the presence of fortified rice in the diet (15 % rather than 5 %), we see much larger increases in Fe PA on average in the full AHS sample (women 77 %; children 87 %) and especially in rice-buying AHS households (women 95 %; children 99 %).

Increases in MPA with fortification of rice were constrained by deficient nutrients that are not included in the fortificant premix such as Ca, vitamins B_2_ (riboflavin) and C. In rice-purchasing households, MPA increased by around 30 percentage points (pp) for all groups, to around 70 % for women and children and 80 % for men.

We assessed risk of exceeding upper tolerable intake levels (UL) and found no issues. We however noted that, to avoid exceeding UL for niacin, niacinamide will need to be used (as required by the WFP specification) in fortification because it has a much higher UL (900 mg/d) than nicotinic acid (35 mg/d). However, for populations reliant on groundwater such as those in the *Terai*, our estimates of Fe adequacy are uncertain due to the wide variance in estimates of Fe concentrations in groundwater in Nepal^(68–71)^. Sensitivity analyses of adding the lowest estimate of 0·04 mg/d^(70)^ and a water intake of 2 l/d^(72)^ would result in almost zero change in PA estimates. Adding a very high estimate (42 mg/d) as found in Bangladesh^(73)^ would result in a PA from water intakes alone of 1 for pregnant women, men and women if 15 % bioavailability is assumed. With 5 % bioavailability, all pregnant women and men would have an Fe PA of 1 and non-pregnant women a PA of >0·85.

The risk of exceeding UL of Fe would be high if consuming 42 mg/d from water, since the upper level cut-off is 45 mg/d. However, the probability of such high intakes is low, since Nepal estimates include 0·05–10·81 mg/l (eastern Terai^(68)^), 0·3 to 19·5 mg/l (central Terai^(71)^) and 0·02 to 1·9 mg/L^(69,70)^ (Kathmandu valley). Therefore, although Fe contamination of water is more likely in the *Terai*, more rice is grown so people are less likely to consume fortified rice in this region. Nevertheless, monitoring of Fe contamination, water intakes and serum ferritin levels may need to accompany implementation of rice fortification programmes going forward.

**Fig. 4:**
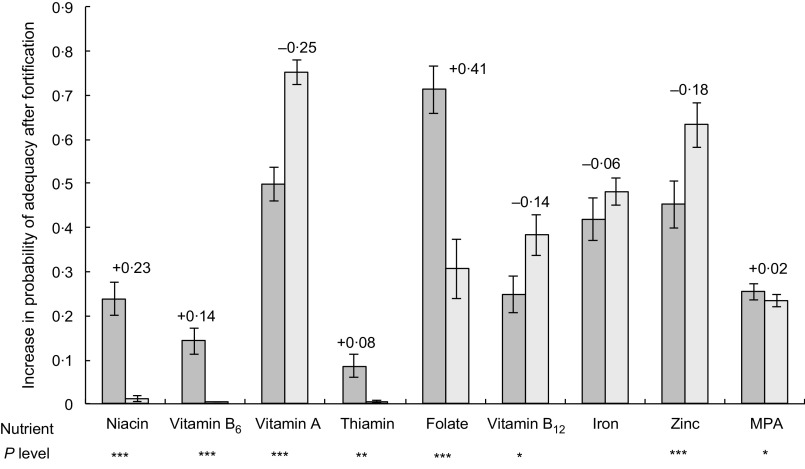
Comparison of the difference in probability of adequacy (PA) with and without fortification, between pregnant women and male household heads. Values given above the bars for women represent the difference between pregnant women’s and men’s increase in PA after fortification of rice. *P* values for tests of differences in the increase in PA between pregnant women and men are provided below each nutrient name, where * *P* < 0·05, ** *P* < 0·01, *** *P* < 0·001. 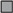, women; 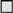, men. MPA, mean probability of adequacy

Analysis of the impact of fortification on gender equity (Fig. 4) shows that women benefit from fortification relatively more than men.

Increases in adequacy were significantly higher in women than in men for vitamins B_9_ (folate) (41pp; *P* < 0·001), B_3_ (niacin) (23pp; *P* < 0·001), B_6_ (pyridoxine) (14pp; *P* < 0·001), B_1_ (thiamin) (8pp; *P* < 0·01) and overall MPA (2 pp; *P* < 0·05), whereas for vitamins A, B_12_ and Zn, increases were significantly higher for men (25, 14 and 18 pp respectively), and increase in Fe adequacy was similar.

Regional differences in potential impact of fortification are shown in Table 4.

**Table 4:** Regional comparisons of the difference in women’s mean probability of adequacy, with and without fortification (*n* 5443)*

	Mean	sd	Conditional mean estimates	95 % CI	*P*
Federal State of Nepal					
Province 3	0·17	0·16	Ref		
Province 1	0·17	0·18	0·00	−0·05, 0·05	0·977
Province 2	0·11	0·15	−0·06	−0·11, −0·01	0·013
Province 4	0·13	0·15	−0·04	−0·09, 0·00	0·077
Province 5	0·15	0·15	−0·02	−0·09, 0·05	0·575
Province 6	0·21	0·15	0·04	0·00, 0·07	0·068
Province 7	0·12	0·17	−0·05	−0·12, 0·02	0·127
Ecological zone					
Terai	0·11	0·15	Ref		
Hill	0·19	0·17	0·08	0·05, 0·10	<0·001
Mountain	0·19	0·16	0·07	0·01, 0·14	0·019
Social safety net areas					
Non-NFC	0·14	0·16	Ref		
NFC district	0·20	0·17	0·05	0·01, 0·10	0·012
Rural/urban					
Urban	0·18	0·19	Ref		
Rural	0·13	0·15	−0·05	−0·09, −0·01	0·011

NFC, Nepal Food Corporation.

* Fortification using World Food Programme specifications using the third Nepal Annual Household Survey data.

We find minimal differences across provinces, but significantly larger improvements in MPA in hills (8pp; *P* < 0·001) and mountains (7pp higher; *P* < 0·05) than the plains and 5pp (*P* < 0·05) lower improvements in rural than urban areas.

Our test of agreement between observed intakes and intakes predicted by the application of AME (see online supplementary material, Supplemental Table 3) indicates that the application of AME does not result in large bias (range −351 to 276 kJ) but does overestimate pregnant women’s intakes, underestimate men’s intakes and gives wide limits of agreement. This overestimation is greatly increased if AME account for pregnancy status. Incorporation of self-reported activity levels to adjust AME also does not appear to increase agreement between observed and predicted intakes.

## Discussion

This is the first study to model the potential effects of rice fortification on probability of micronutrient adequacy and to examine its equity implications. We find that Nepalese unfortified diets are inadequate, and rice is consumed in high quantities, making rice suitable for fortification. Nutrient inadequacy is high for vitamins B_12_, A, B_9_ (folate), Fe and Zn (all potentially fortifiable in rice) and also Ca and riboflavin. Different geographical areas are likely to benefit differentially from rice fortification, depending upon where purchased rice consumption is highest (remote province 6, the mountains and urban areas). Perhaps unsurprisingly, we find that replacing normal purchased dehulled, not-parboiled polished white rice with fortified rice (as per the mid-point between minimum and maximum WFP specifications) would resolve dietary deficiencies in rice-purchasing households amongst all population subgroups analysed for all fortified micronutrients, except vitamin B_12_ and Fe in pregnant women (if higher bioavailability is assumed). However, the equity implications are arguably more interesting and important. Rice fortification is predicted to increase pregnant women’s adequacies more than adult males for most nutrients. Due to higher intakes of bought rice, the nutritional adequacies of more remote hilly and mountainous populations are predicted to improve more in response to rice fortification than in the plains, as will adequacy in urban compared with rural areas.

### Implications for addressing micronutrient deficiencies

Our results indicate that WFP 2016 specification levels for rice fortification are generally appropriate and could address major nutrient gaps, although we could consider reducing levels of B_3_ (niacin), which is already found in rice. For a location where rice consumption is 150–300 g/capita per d, de Pee^(46)^ and de Pee *et al.* ^(49)^ recommended the following fortification levels: Fe (micronised ferric pyrophosphate), 7 mg/100 g (or if ferric pyrophosphate is combined with citrate and trisodium citrate, or possibly other solubilising agents 4 mg/100 g); folic acid (B_9_) 0·13 mg/100 g; vitamin B_12_ (cyanocobalamin) 0·001 mg/100 g; vitamin A (palmitate) 0·15 mg/100 g; Zn (oxide) 6 mg/100 g; B_1_ (thiamine mononitrate) 0·5 mg/100 g; B_3_ (niacin amide) 7 mg/100 g and B_6_ (pyridoxine hydrocloride) 0·6 mg/100 g. The WFP 2018 updated recommendation provides corresponding increases/decreases per 100 g with decreasing/increasing levels of rice consumption^(49)^. We found that the 2016 median WFP specification we tested broadly matched these new recommendations but had slightly elevated levels of most micronutrients.

For pregnant women, additional Fe and Vitamin B_12_ from improved dietary quality and supplements would still be required to reach adequacy. Since the Nepal government distributes free Fe/folic acid supplements for 225 d of pregnancy and early lactation, the shortfall in these micronutrients should be met amongst pregnant and lactating women who consume these supplements sufficiently. However, vitamin B_12_, which is only found in animal-source foods, appears to be a problem nutrient, especially in the plains.

Although riboflavin (B_2_) intakes are inadequate, we do not suggest adding riboflavin to the Nepal fortificant premix, due to colour changes in the fortified rice kernels which reduce consumer acceptability^(63)^. Whilst levels of fortificants recommended in fortified rice are safe, caution has been advised due to an association between fortified rice consumption and hookworm infection^(74)^. Promotion of fortified rice, especially in schools, needs to be accompanied with deworming and public awareness campaigns promoting the use of footwear and other hygiene and sanitation practices.

Micronutrient deficiencies in the Nepalese diet we investigated are similar to those found in mothers and young children in Bangladesh, although vitamins A, B_9_ (folate) and Zn deficiencies were worse and vitamin B_12_ less severe in Bangladesh^(75)^. Our estimates suggest higher adequacy in pregnant women and other population subgroups in 2015 than those found for lactating mothers in urban Nepal in 2009^(9)^. Nevertheless, the diets we studied are severely deficient, and staple fortification is warranted.

One study applied a similar modelling approach to ours using UK dietary consumption data. They simulated vitamin D fortification of wheat flour and milk and analysed the effect upon adequacy of intake amongst at-risk population subgroups. When wheat flour was fortified, the proportion estimated to have vitamin D intakes below reference nutrient intakes fell from 93 to 50 % without any individual exceeding UL^(76)^. In Vietnam, Laillou *et al.* ^(77)^ similarly used data from a national survey of women’s diets to estimate the impact of different fortified foods upon dietary intake. Rice fortified with Fe, Zn and folic acid increased intakes as a percentage of the reference nutrient intake by 41 % for Fe, 16 % for Zn and 34 % for B_9_ (folate), making fortified rice the most appropriate fortification vehicle in that setting^(77)^. In 2013, a Fortification Assessment Coverage Toolkit was designed to help stakeholders collect, analyse and synthesise standardised data on quality, coverage and consumption of fortified foods. By assessing amounts of fortifiable and fortified foods consumed, ‘feasibility gaps’ and ‘fortification ‘gaps’ can be quantified to estimate the potential of different fortified foods and the extent it is being fulfilled^(34)^. The limitation in this method is that it ignores other (non-fortified) dietary sources, a problem which is rectified in our approach.

The potential clinical significance of the increases in micronutrient adequacy that could result from the consumption of fortified rice is wide-ranging and includes: improved Fe^(25)^, Zn^(78)^, vitamin B_12_^(79)^ and vitamin A status^(23,80)^; lower prevalence of anaemia^(25,26,30,81,82)^, beriberi^(83)^ and neural tube defects^(84)^ and improved cognition^(22)^ and physical performance^(78)^.

### Implications for reaching the poorest

Many farmers grow and consume their own rice, and it is not currently feasible to blend fortified rice kernels with home-grown rice in small-scale mills, so only rice milled commercially in large mills will be fortifiable in the foreseeable future, and only rice-buyers will benefit^(33)^. We found rice buyers across the wealth spectrum in urban areas (including poorer and wealthier households) and in hilly and mountainous areas with low rice production. Additional interventions are needed for households who do not buy much rice, especially in *Terai* areas where both home production and micronutrient deficiencies are highest^(43)^.

Of those who do purchase rice, fortification may benefit better-off households most. Voluntary fortification is likely to precede or preclude mandatory fortification, and the incremental price increase will likely deter poorer buyers from choosing it^(85)^. Also, the private sector may prefer to fortify and market more expensive rice varieties and market its higher nutritional value, which would only be afforded by better-off consumers. One potential means by which rice fortification can benefit those who need it most will be to distribute fortified rice via social safety nets^(85)^ as per the Government of Nepal’s plan. If subsidised fortified rice is distributed through the NFC, it could reach the most food-insecure and inaccessible areas of the country. However, to reach the poorest elsewhere, wider access to low-cost or subsidised fortified rice will be needed.

We examine the potential for rice fortification to be a gender-sensitive intervention. We find that pregnant women in province 2 are likely to see bigger improvements in their micronutrient adequacy from rice fortification than men, which could help address the gender gap in micronutrient adequacy. This may be because women tend to rely heavily on rice to meet their dietary needs^(8,9,11)^, and their diets comprise a higher share of starchy staples than men’s^(39)^, but also their diets are more deficient, so gains are easier to achieve. Of course, rice fortification is not a replacement for women’s empowerment or behaviour change interventions aiming to improve women’s dietary quality and equity of nutrient intake within households, but it would complement such interventions.

### Study limitations

Limitations of our study include extrapolation to the entire population, whereas in the short to medium term, only a small proportion of the population who purchase NFC social safety net rice and WFP school meals programme recipients would actually benefit. To realise predicted benefits, households would need all their purchased rice to be fortified. Families are likely to substitute unfortified home-grown rice with bought, fortified rice when their grown rice has run out. This means there may be benefits during the lean season before rice harvest but less benefit post-harvest in the winter when fruits/vegetables availability is limited at high altitudes. Having said this, the pre-harvest lean period is when additional intakes through fortification are most needed because multiple micronutrient serum concentrations (especially β-carotene, B_6_, B_9_ (folate) and Fe concentrations) are notably low^(6)^.

In LBWSAT, the share of rice that was purchased was estimated by ranking the relative importance of purchase to meet staple food needs. Use of this rule of thumb to assume the proportion of rice purchased is a source of error that we cannot quantify. Both LBWSAT and AHS III surveys were collected over several months (June–September 2015 for LBWSAT and September 2014 to July 2015 for AHS III) but neither covered 12 months. This means that seasonal variation outside the periods sampled is not accounted for in our findings.

We have estimated mean intakes for men, non-pregnant women and children in the AHS III using AME to allocate nutrients to household members as per energy requirements. The assumption that women are not pregnant is important because Bland–Altman limits of agreement show that AME overestimate the intakes of pregnant women in particular. The AHS III dataset was missing eggs and green leafy vegetables, and a relatively short list of sixty foods were reported, so adequacy may be underestimated. However, conservative sensitivity analyses, and comparison with LBWSAT results, show the same key trends: adequacy of diets drastically improves with fortification but vitamin B_12_ (and Fe for pregnant women) remains problem nutrients, and MPA is constrained by unfortified nutrients such as riboflavin (B_2_), Ca and vitamin C.

Our estimates are based upon uncooked rice nutrient values which have not been adjusted for losses during cooking. Hence, there is some risk of overestimating intakes of vitamins B_1_, B_2_ and to a lesser extent B_9_. However, our intake estimates from uncooked rice from are very similar to those from a slightly larger LBWSAT sample which used cooked rice values^(39)^, indicating that the error introduced from using uncooked rice is small.

The LBWSAT dietary adequacy estimates for pregnant women and their families may not be generalisable to more wealthy and non-plains populations of Nepal, since Dhanusha and Mahottari districts fall are relatively poor and inequitable intra-household food allocation is particularly prevalent^(8,39)^. On the other hand, since the AHS III is a nationally representative survey, the findings from the current study are generalisable to Nepal^(42)^. This means that the risk of exceeding UL is low even in wealthier households, except for Fe in scenarios where all rice consumed is fortified, pregnant women take daily Fe supplements and there is high contamination of ground water with Fe.

Future research documenting individual dietary data from different age–sex groups across Nepal would help obtain a more precise estimate of the broader potential impact on nutritional adequacy. Our results are from modelled scenarios, based on assumptions about consumption levels, requirements (infection, activity levels, nutrient retention) and interactions between nutrients. Further research to measure these could inform future models and improve their accuracy. Rather than conducting more trials under experimental conditions, we recommend careful evaluation of rice fortification through NFC and/or the School Meals Programme in Nepal, particularly effects on uptake by the private sector, wider dietary changes, biochemical indicators of micronutrient levels (especially niacin), children’s health and school performance.

We conclude that fortification of rice using the WFP specification, in combination with other health and nutrition programmes, presents an opportunity to contribute to the reduction of micronutrient deficiencies and also to reduce inequalities in micronutrient allocation in Nepal.

## Supplementary material

For supplementary material accompanying this paper visit https://doi.org/10.1017/S1368980020001020. click here to view supplementary material
