# Supplementary material for: Equity implications of rice fortification: a modelling study from Nepal
Source: Public Health Nutr. 2020 Jul 6;23(15):2824–39. doi: 10.1017/S1368980020001020 (PMC7477371; doi:10.1017/S1368980020001020)
Supplement: Supplementary file 1 [file S1368980020001020sup.zip › S1368980020001020sup002.docx]

Supplemental Table 1. Daily frequency of consumption of rice dishes, other staple and common foods ^a^

|  | **Frequency of intakes per day (Mean, *SD*)** | | | | | | | |
| --- | --- | --- | --- | --- | --- | --- | --- | --- |
|  | **Pregnant women** | | **Mothers-in-law** | | **Household heads** | | **Average** | |
|  | Mean | *SD* | Mean | *SD* | Mean | *SD* | Mean | *SD* |
| **Dishes made with polished white rice** |  |  |  |  |  |  |  |  |
| Boiled white rice | 2.03 | *0.6* | 1.74 | *0.54* | 1.72 | *0.67* | 1.83 | *0.62* |
| Fried rice | 0.01 | *0.05* | 0.01 | *0.05* | 0 | *0.04* | 0.01 | *0.04* |
| Rice pudding (*kir*) | 0.03 | *0.12* | 0.05 | *0.14* | 0.02 | *0.08* | 0.03 | *0.11* |
| Rice gruel (*jaulo* /*khichadi*) ^b^ | 0.03 | *0.14* | 0.04 | *0.2* | 0.04 | *0.19* | 0.04 | *0.18* |
| **Other rice-based foods** |  |  |  |  |  |  |  |  |
| Puffed rice | 0.1 | *0.24* | 0.09 | *0.21* | 0.14 | *0.31* | 0.11 | *0.26* |
| Beaten rice | 0.08 | *0.2* | 0.05 | *0.14* | 0.16 | *0.29* | 0.1 | *0.22* |
| Rice flour-based snacks ^c^ | 0.04 | *0.13* | 0.04 | *0.16* | 0.04 | *0.15* | 0.04 | *0.15* |
| **Other starchy staples** |  |  |  |  |  |  |  |  |
| Fresh corn on the cob/ roasted | 0.02 | *0.12* | 0.01 | *0.09* | 0.01 | *0.09* | 0.01 | *0.1* |
| Breads, including flatbreads ^d^ | 1.03 | *0.59* | 0.95 | *0.58* | 0.99 | *0.64* | 0.99 | *0.61* |
| *Haluwa* stiff porridge ^e^ | 0.04 | *0.12* | 0.04 | *0.12* | 0.04 | *0.13* | 0.04 | *0.12* |
| Snacks ^f^ | 0.16 | *0.38* | 0.15 | *0.33* | 0.18 | *0.4* | 0.16 | *0.37* |
| Tubers ^g^ | 0.52 | *0.51* | 0.42 | *0.48* | 0.43 | *0.45* | 0.46 | *0.48* |
| **Other common foods** |  |  |  |  |  |  |  |  |
| Spiced lentil soup - *dal* | 1.06 | *0.64* | 0.9 | *0.55* | 0.92 | *0.55* | 0.96 | *0.59* |
| Tea with sugar and milk | 0.16 | *0.37* | 0.3 | *0.54* | 0.49 | *0.64* | 0.32 | *0.54* |
| Pointed gourd curry | 0.41 | *0.56* | 0.37 | *0.53* | 0.39 | *0.5* | 0.39 | *0.53* |
| Mango ^h^ | 0.3 | *0.48* | 0.33 | *0.45* | 0.28 | *0.41* | 0.3 | *0.45* |
| Buffalo milk | 0.28 | *0.43* | 0.1 | *0.23* | 0.21 | *0.45* | 0.19 | *0.39* |

^a^ *n*=150 from LBWSAT (Low Birth Weight South Asia Trial) sample

^b^ *jaulo* made with rice; *khichadi* made with rice, dal and sometimes vegetables

^c^ Rice flour-based snacks including *sel roti,* *malpuwa* and *bagiya*

^d^ Breads, including flatbreads without oil *roti*, flatbreads made with oil (*puri, paratha, naan*) and risen bread (*pau roti*)

^e^ *haluwa* stiff porridge made with flour (various types of staples), oil, and sugar

^f^ Snacks: starchy fried &/or sweet snacks

^g^ Tubers: boiled, fried, in curry or in chutney

^h^ Data were collected during June – September. Peak mango season is June to July.

**Supplemental Table 2. Mean dietary intakes of micronutrients in Bangladesh rice premix**^a^ **after fortification of purchased rice**

|  | **National AHS III** | | | | |  | | **LBWSAT** | | | | | | | | | |  | |  |  |
| --- | --- | --- | --- | --- | --- | --- | --- | --- | --- | --- | --- | --- | --- | --- | --- | --- | --- | --- | --- | --- | --- |
|  | Women  15-49y | | | Children  5-12y | | | | Pregnant women | | | | Mothers-in-law | | | | Male HH heads | | | |  |  |
| **Sample size** | ***n*** |  | | ***n*** | |  | | ***n*** | |  | | ***n*** | |  | | ***n*** | |  | |  |  |
| Fortified (all)^b^ | 5443 |  | | 3346 | |  | | 128 | |  | | 128 | |  | | 128 | |  | |  |  |
| Fortified (buyers)^c^ | 3007 |  | | 1882 | |  | | 97 | |  | | 97 | |  | | 97 | |  | |  |  |
| **Nutrient intakes** | mean | *SD* | | mean | | *SD* | | mean | | *SD* | | mean | | *SD* | | mean | | *SD* | |  |  |
| Vitamin A (RE/d) |  |  | |  | |  | |  | |  | |  | |  | |  | |  | |  |  |
| Fortified (all) | 615 | *576* | | 490 | | *444* | | 779 | | *525* | | 824 | | *771* | | 860 | | *489* | |  |  |
| Fortified (buyers) | 859 | *537* | | 677 | | *413* | | 865 | | *549* | | 903 | | *834* | | 936 | | *485* | |  |  |
| Vitamin B_1_ (mg/d) |  |  | |  | |  | |  | |  | |  | |  | |  | |  | |  |  |
| Fortified (all) | 2.5 | *1.2* | | 2 | | *1.1* | | 2.6 | | *1* | | 2.6 | | *1.1* | | 3.3 | | *1.2* | |  |  |
| Fortified (buyers) | 3.2 | *1.3* | | 2.6 | | *1.1* | | 2.9 | | *1.1* | | 2.9 | | *1.1* | | 3.6 | | *1.2* | |  |  |
| Vitamin B_9_ (μg/d) |  |  | |  | |  | |  | |  | |  | |  | |  | |  | |  |  |
| Fortified (all) | 685 | *528* | | 562 | | *450* | | 768 | | *409* | | 770 | | *400* | | 912 | | *426* | |  |  |
| Fortified (buyers) | 1086 | *454* | | 885 | | *396* | | 904 | | *373* | | 894 | | *367* | | 1056 | | *374* | |  |  |
| Vitamin B_12_ (μg/d) | | |  | |  | |  | |  | |  | |  | |  | |  | |  | | |
| Fortified (all) | 2.4 | *2.4* | | 2 | | *2* | | 2.7 | | *1.9* | | 2.6 | | *3.1* | | 3.1 | | *2.1* | | |  |
| Fortified (buyers) | 4.2 | *2* | | 3.5 | | *1.7* | | 3.3 | | *1.7* | | 3.3 | | *3.2* | | 3.9 | | *1.8* | | |  |
| Iron (mg/d) |  |  | |  | |  | |  | |  | |  | |  | |  | |  | | |  |
| Fortified (all) | 21.1 | *12.7* | | 17.4 | | *10.9* | | 24.9 | | *10.4* | | 24.9 | | *11.6* | | 31.3 | | *11.4* | | |  |
| Fortified (buyers) | 29.9 | *11.8* | | 24.5 | | *10.1* | | 27.7 | | *10.1* | | 27.9 | | *11.3* | | 34.7 | | *10.5* | | |  |
| Zinc (mg/d) |  |  | |  | |  | |  | |  | |  | |  | |  | |  | | |  |
| Fortified (all) | 15.8 | *8.8* | | 13 | | *7.7* | | 16.7 | | *7.2* | | 16.5 | | *7.7* | | 20.6 | | *8* | | |  |
| Fortified (buyers) | 21.7 | *8.6* | | 17.8 | | *7.4* | | 18.6 | | *7.1* | | 18.5 | | *7.5* | | 23 | | *7.3* | | |  |

Male HH heads: male household heads

^a^ using Bangladesh standards

^b^ Fortified (all): intakes of total population when bought rice is fortified with Bangladesh premix (mid-values of household fortificant levels rather than factory levels);

^c^ Fortified (buyers): intakes of rice-buying households only, when bought rice is fortified with Bangladesh premix (mid-values of household fortificant levels rather than factory levels)

**Supplemental Table 3. Agreement between observed and predicted daily energy intakes**^1^

|  | **Agreement between observed and predicted daily energy (kJ) intakes from LBWSAT** | | | | | | | | | | | | | | | | |
| --- | --- | --- | --- | --- | --- | --- | --- | --- | --- | --- | --- | --- | --- | --- | --- | --- | --- |
|  | **Pregnant women^2^** | | | | | **Mothers-in-law^2^** | | | | | | **Male household head^2^** | | | | | |
| **Adult male equivalents assumptions** | Mean kJ differ-ence | (95% CI) | | LOA | | Mean kJ differ-ence | (95% CI) | | LOA | | Mean kJ differ-ence | | (95% CI) | | LOA | |  |
| Moderate PAL no adjustment ^3^ | -351 | (-649, | -54) | -4050 | 3347 | 75 | (-180, | 335) | -3130 | 3284 | 276 | | (-63, | 611) | -3916 | 4464 |  |
| Moderate PAL with adjustment ^4^ | -1301 | (-1607, | -996) | 0 | 2477 | 498 | (234, | 761) | -2766 | 3761 | 803 | | (469, | 1142) | -3368 | 4975 |  |
| Self-reported PAL^5^ no adjustment^3^ | -33 | (-322, | 259) | -3623 | 3561 | -138 | (-397, | 121) | -3364 | 3088 | 172 | | (-163, | 502) | -3929 | 4272 |  |
| Self-reported PAL^5^ with adjustment^4^ | -1004 | (-1301, | -711) | 0 | 2632 | 297 | (33, | 561) | -2946 | 3540 | 707 | | (381, | 1038) | -3360 | 4774 |  |
| LOA = Limits of agreement, calculated as the mean difference ± 1·96 SD;  PAL = Physical Activity Levels. ^1^ from LBWSAT data, intakes in kJ; ^2^ n=150; ^3^ no adjustment for pregnancy; ^4^ with adjustment for pregnancy;  ^5^ Based on self-reported activity levels from 24-h recall period, categorised as sedentary (PAL=1.6); moderate (PAL=1.9); strenuous (PAL=2.2) and weight (kg) | | | | | | | | | | | | | | | | | |
